# Optimisation of alpha-amylase inhibitor production in solid state fermentation

**DOI:** 10.3389/fphar.2023.1073754

**Published:** 2023-03-22

**Authors:** O. A. Fatoki, A. A. Onilude, Y. A. Ekanola, C. T. Akanbi

**Affiliations:** ^1^ Department of Biology, The Polytechnic, Ibadan, Nigeria; ^2^ Department of Microbiology, University of Ibadan, Ibadan, Nigeria; ^3^ Department of Food Science and Technology, Obafemi Awolowo University, Ile-Ife, Nigeria

**Keywords:** alpha-amylase inhibitor, optimisation, RSM, *Streptomyces*, solid state fermentation

## Abstract

Though not a known producer of alpha-amylase inhibitor, the potential of *Streptomyces xinghaiensis* AAI-2 to produce this important metabolite was assessed and the process optimised in solid substrate using response surface methodology. The isolate was grown in an inoculum medium, inoculated into wheat bran and supplemented with a basal medium for production of alpha amylase inhibitor. Optimum conditions were determined by Response Surface Methodology. The extract was recovered using sodium phosphate buffer at refrigerated temperature and assay for the presence of alpha-amylase inhibitor was carried out by Dinitrosalicylic acid method. Based on the results of the experimental trials and iteration with those values, it was predicted that optimal pH for alpha-amylase inhibitor production using *S. xinghaiensis* in solid culture of wheat bran was pH 6.4–6.9 while optimal moisture content and incubation time were predicted as 71%–73% and 9–12 days respectively.

## Introduction

Alpha-amylase inhibitors are glycoproteins that can inhibit starch hydrolysis by alpha-amylase. This hydrolysis yields simpler, less complex subunits by hydrolysing the α-1, 4 bonds of starch at its non-reducing end. Due to the presence and role of this enzyme in the digestive tract of some animals including man, alpha-amylase inhibitors (αAI) have been proposed to be useful in the regulation of the rate of digestion of starch. From its discovery by Marshall and Lauda (1975) as a source of an amylase inhibitor, white kidney bean (*Phaseolus vulgaris*) is arguably the most widely studied source of the alpha amylase inhibitor. Related research has involved purification and the elucidation of characteristics, application and clinical trials, expression of gene in competent microorganisms, uses as nutraceutical agent among others, including a more recent publication on the incorporation of *P. vulgaris* in yoghurt for the control of blood glucose in hyperglycemic mice *via* the modulation of microorganisms in the gastrointestinal tract (Wang et al., 2021). While numerous plant sources exist and have been identified, synthetic types including miglitol, acarbose, voglibose and indazoles also exist (DiNicolantonio et al., 2015; Garg et al., 2022). Submerged fermentation of filamentous bacteria of the genus *Streptomyces* has also yielded alpha-amylase inhibitors which have been variously characterized and applied (Manivasagan et al., 2015). The advantages associated with solid state fermentation include secretion of metabolites that are better adapted to numerous environmental conditions and applications (Leite et al., 2021). Furthermore, the process is considered cheaper, since the carbon and energy sources for microbial use are mostly agricultural wastes. Since the need for water is less, there is a consequent low effluent produced and a reduced need to use water treatment processes when done on an industrial scale (Holker and Lenz, 2005). The low water activity is thought to discourage the growth of contaminating yeast and bacteria thereby minimising the strict need for sterilisation, hence energy. The optimal conditions for production of αAI in solid production medium using actinomycetes was investigated.

## Materials and methods

### Isolation and identification

Soil samples collected from 10 cm depth of an area (7.44°N, 3.89°E) within the premises of University of Ibadan, Nigeria having active plant degradation was pre-treated by air drying for 24 h. Further pre-treatment was done by subjecting a suspension of the pre-treated soil to heat at 70°C for 15 min and subsequently adding 1.5% phenol (v/v) to the soil suspension (Seong et al., 2001; Tiwari and Gupta, 2012). A ten-fold serial dilution of the soil suspension was done and 0.1 mL of the diluted sample was inoculated in sterile starch casein nitrate agar (g/L): [starch (10), casein (0.3), KNO_3_ (2.0), NaCl (2.0), K_2_HPO_4_ (2.0), CaCO_3_ (0.02), MgSO_4_.7H_2_O (0.05), FeSO_4_.7H_2_O (0.01), agar (15)] and supplemented with nystatin (50 μg/mL) and nalidixic acid (20 μg/mL) as antifungal and antibacterial agents respectively. The culture was incubated for 10 days at 28°C.

Isolates were characterised by observation of morphological characteristics, biochemical tests (Nonomura, 1974) and molecular identification.

### Production of alpha-amylase inhibitor

Wheat bran was air-dried to constant weight and sterile basal medium (cornsteep liquor (0.4% v/v), glucose (1% w/v), (NH_4_)_2_HPO_4_ (0.8% w/v), soyflour (0.4% w/v), peptone (1% w/v) at pH 8.3) was introduced and the set up was sterilized. *Streptomyces* inoculum was prepared in the following medium: [glucose (1%), soy flour (1%) and NaCl (0.25%)] (Oeding et al., 1980). The inoculum was introduced into sterile solid substrate and incubated at 28°C ± 2°C for production of αAI.

### Optimisation of production conditions

Optimum conditions for the production of αAI were determined by the Response Surface Methodology (RSM). Three independent parameters; moisture content (%), pH and period of incubation (days) were varied at three levels (−1, 0, +1), using the Box–Behnken design. The conditions were varied as follows: moisture content (65%, 70%, 75%), pH (6.0, 7.0, 8.0) and incubation period (5 days, 10 days, 15 days). The variables were set up for 17 experimental runs (Table 1).

**TABLE 1:** Box—Behnken design matrix for optimisation of pH, moisture content and incubation time for alpha–amylase inhibitor production by actinomycetes.

Coded variables			
Trials	pH	Moisture	Time
1	−1	−1	0
2	+1	−1	0
3	−1	+1	0
4	+1	+1	0
5	−1	0	−1
6	+1	0	−1
7	−1	0	+1
8	+1	0	+1
9	0	−1	−1
10	0	+1	−1
11	0	−1	+1
12	0	+1	+1
13	0	0	0
14	0	0	0
15	0	0	0
16	0	0	0
17	0	0	0

### Extraction and purification

Recovery of the αAI from the *Streptomyces* culture on wheat bran was carried out according to the method of Mulimani and Supriya (1993) with modifications. At refrigerated temperature, using sodium phosphate buffer (20mM; pH 6.9), the extract was recovered in a centrifuge at 10,000×g for 20 min at 4°C. The supernatant was decanted and stored as crude alpha-amylase inhibitor extract. Further purification was done by ammonium sulphate precipitation, overnight dialysis against extraction buffer at 4°C and column chromatography using Sephadex G-100.

### Assay

The presence of αAI in the extract was determined by the Dinitrosalicylic acid (DNSA) assay method described by Bernfeld (1955). Readings were taken at 540 nm using a UV-Visible Spectrophotometer.

## Results

**FIGURE 1:**
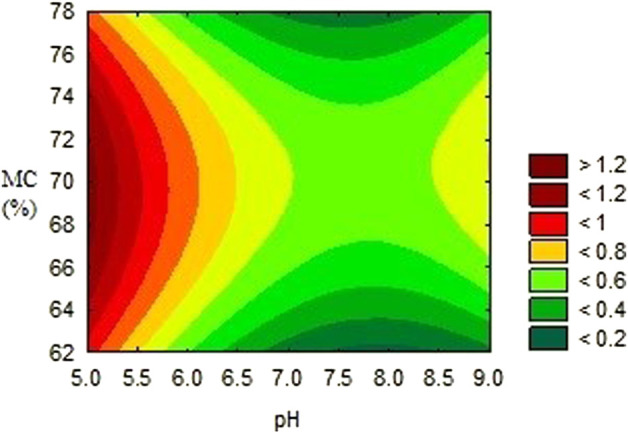
Effects of moisture content and pH on the activity of alpha–amylase inhibitor.

The isolate obtained was identified with a grey aerial mycelium, which appeared powdery with a firm adhesion to the agar surface. It could utilise all Carbon sources tested except inositol and could grow in the presence of NaCl up to 10%. Moreover, the isolate could grow in the presence of citrate but not in the presence of urea (Table 2). Molecular identification using the partial sequence of the 16S rRNA identified the isolate as *Streptomyces xinghaiensis* (GenBank accession number—KY858944). The isolate was observed to fall into the same sub-clade with *S. rubrus* and *S. tendae* which have 98% and 97% similarity respectively with the isolate (Figure 1).

**TABLE 2:** Characteristics of *S.xinghaiensis* AAI-2.

Test/Characteristics	Result
Colour of aerial mycelium	Grey
Appearance	Powdery
Colour on reverse side	Cream

Biochemical Characteristics	
Carbon Utilisation	
Inositol	Negative
Sucrose	Positive
Lactose	Positive
Maltose	Positive
Mannitol	Positive
Sorbitol	Positive
Growth In NaCl (2%–10%)	Positive
Citrate utilisation	Positive
Urea hydrolysis	Negative

**FIGURE 2:**
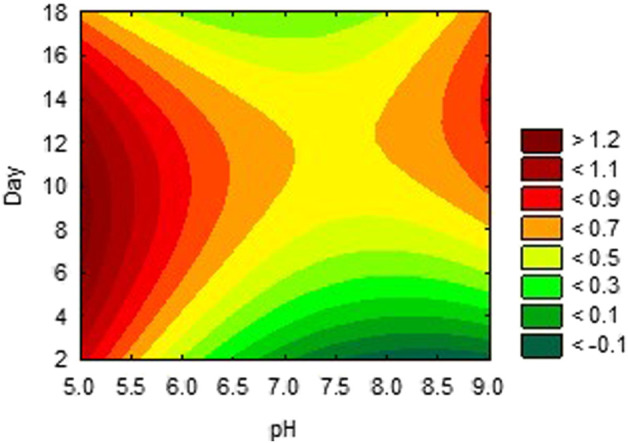
Effects of incubation period and pH on activity of alpha-amylase inhibitor.

**FIGURE 3:**
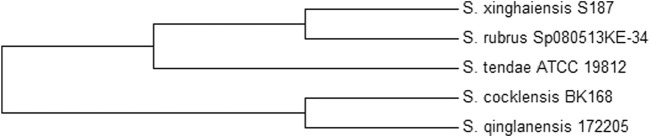
Phylogenetic tree showing the position of *S. xinghaiensis* among some closely related species.

**FIGURE 4:**
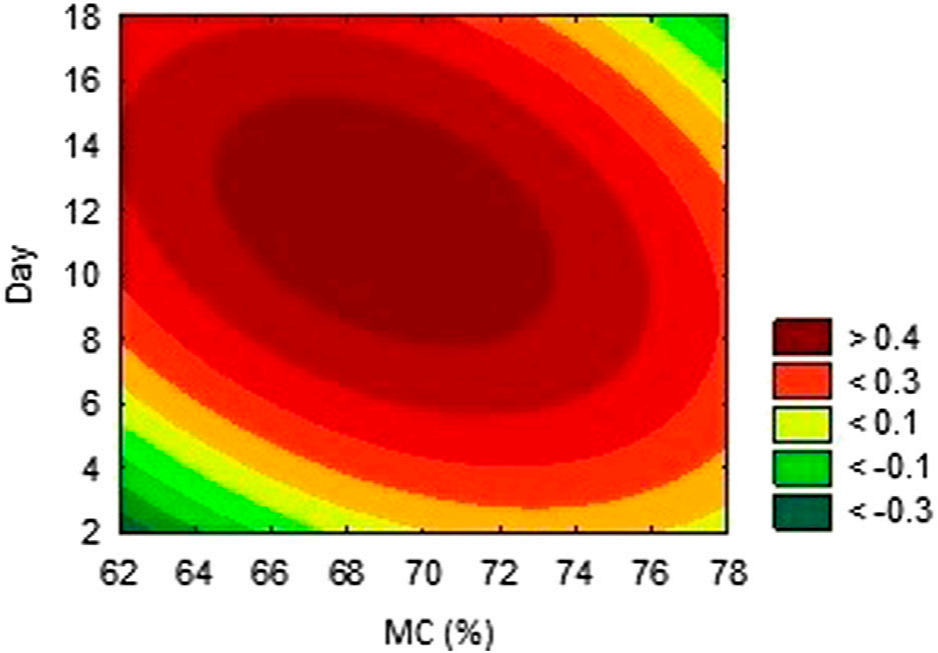
Effects of incubation period and moisture content on the activity of AAI.

The activities obtained from each experimental trial were analysed, in order to obtain the optimum conditions of each environmental condition. The results are presented in Figures 2, 3, 4. These results were further iterated and the optimum values were predicted as shown in Table 3. The results show that optimum production conditions were obtained at 6.38–6.87 pH, 70.7%–73.4% moisture content and 8.5–12 days of incubation.

**TABLE 3:** Predicted optimum values of the three variables.

Fermentation conditions	Predicted optimised values				
pH	6.375710	6.740031	6.871774	6.482091	6.668138
Moisture (%)	70.73637	72.02184	72.46204	73.00302	73.42530
Time (Days)	11.93176	9.19989	8.51072	9.75152	8.80241

## Discussion

*Streptomyces xinghaiensis* AAI2 isolated from soil was positive for starch, citrate and casein hydrolysis as well as the utilisation of many carbon sources for growth except inositol. These group of organisms are known to be able to degrade various kinds of substrates usually due to their ability to secrete a wide range of hydrolytic enzymes. This isolate was obtained from an environment characterized by active degradation of plant material; a factor that is capable of contributing to its ability to synthesize hydrolytic enzymes which are required for growth and absorption of nutrients. Citrate hydrolysis was not initially reported in this specie (Zhao et al., 2009; Kumar et al., 2012) as observed in this study. The partial sequence of the isolate bore 97% similarity to the complete genome of a known producer of alpha amylase inhibitor, *S. tendae* which is responsible for the production of the alpha amylase inhibitor named tendamistat (Vertesy et al., 1984). This similarity, in addition to information from the phylogenetic tree may infer relatedness in evolution and the presence of genes responsible for αAI production. Though *S. xinghaiensis* has never been identified as a producer of alpha amylase inhibitor, its metabolic potential tend towards being prolific, which is a characteristic of the genus. It has been shown from the draft genome sequence that this species can produce many new metabolic products. Of the 6,654 coding sequences contained in the genome sequence of *S. xinghaiensis,* 1,091 coding sequences are for proteins which do not match any proteins in the COG reference databases (Zhao and Yang, 2011). Furthermore, a study conducted between 2013 and 2017 discovered 167 new bioactive metabolites from 58 rare actinomycetes; confirming the metabolic dexterity of actinomycetes (Subramani and Sikpema, 2019).

Using the starch hydrolysis test, the isolate was putatively presented as amylase-producing. This ability formed the basis of its selection for αAI production since it has been opined that enzyme inhibitors may be found in the presence of the enzymes that they inhibit (Umezawa, 1973). αAI was produced by the isolate, using solid substrates which acted both as support for growth of the actinomycetes as well as the source of Carbon. The production of enzyme inhibitors in fermentation media is affected by factors including nature of the substrate, its moisture content, nutrient and mineral composition of the medium. Moreover, the importance of trace elements such as Manganese and Magnesium in the fermentation medium for enzyme inhibitor production has been emphasized. Wheat bran has been used as a support as well as nutrient source for the growth of many microorganisms and its success is attributed to the components of wheat bran which includes starch, protein and minerals (Rao et al., 2005; Verni et al., 2019). The ability of *Streptomyces* sp. to secrete extracellular enzymes such as hydrolases which are used to utilise both simple and complex molecules as nutrients makes them able to effectively utilise wheat bran as a solid substrate (Kapur et al., 2018). Wheat bran and the basal medium also contain nitrogen sources which have been found to have significant effects in the secondary metabolism of *Streptomyces* (Dilipkumar et al., 2013). In fact, cornsteep liquor and soybean flour were identified as Nitrogen sources which are necessary in the synthesis of inhibitor by *Streptomyces* (Vertesy et al., 1984). Tan *et al.* (2020) also supported the notion that the inclusion of soybean meal (which contains 49% crude protein, many important amino acids and microelements) in synthetic media has an advantage of enhancing metabolism in *Streptomyces*. Other important factors that determine product formation also include the pH, moisture content and duration of incubation.

The simplex search method of optimising conditions, using the data obtained from laboratory trials predicted the optimum production of αAI, as occurring between 9 and 12 days of incubation. This claim is corroborated by the fact that the production of metabolites occurs after the exponential phase, which lasts for about 3–7 days in actinomycetes, depending on the environmental conditions and the species being used. Metabolite production which is initiated at this stage, is usually triggered by hyphal differentiation, stress or depletion of nutrients in the environment (Yague, et al., 2013; Manteca and Yagüe, 2018). The αAI AI-3688 was formed in culture solution after a maximum of 10 days. Research has reported other secondary metabolites produced by *Streptomyces* are produced in similar duration.

The importance of moisture in SSF and its effect on utilization of substrate for secondary metabolites formation is attributed to interference with physical properties of the solid particles (Nema et al., 2019). In fermentation, wheat bran absorbs liquid, leading to a swelling of the substrate particles, release and dissolution of the nutrients embedded in the substrate, resulting in better utilisation of the substrate by the microorganism (Manan and Webb, 2019). While increase in moisture beyond the optimum may reduce porosity of the substrate and limit oxygen and mass transfer, low moisture content however, causes reduction in nutrient solubility and a low degree of swelling (Pandey, 2003). In similar research optimising metabolite production by *Streptomyces,* Kar *et al.* (2010) found the optimum production of secondary metabolite using *Streptomyces* at 70% moisture content. Tylosin production by a strain of *S. fradiae* and oxytetracycline by *S. rimosus* and *S. vendagensis* in solid substrate were reported to be optimum at moisture content of about 70% (Okorie and Asagbra, 2008; Khaliq et al., 2009).

An initial pH of 6.4–6.7 was predicted as optimum for the production of αAI using RSM. pH of the medium plays an important part in the physiology of the microorganism as it can affect the morphology of the organism as well as production of secondary metabolites (Bajaj and Singh, 2010). The results obtained favourably compares with the results of Vertesy *et al.* (1984) that discovered the production of an alpha–amylase inhibitor at the pH values that favour the growth of *Streptomyces.*

## Conclusion

In addition to the suitability of solid substrate (wheat bran) culture medium and other conditions for production of alpha amylase inhibitor by *S. xinghaiensis* AAI2, this novel producer of alpha amylase inhibitor within the *Streptomyces* genus, had optimum production conditions for pH, moisture content and incubation period at 6.4–6.9, 71%–73% and 9–12 days respectively.

## Data Availability

The datasets presented in this study can be found in online repositories. The names of the repository/repositories and accession number(s) can be found below: https://www.ncbi.nlm.nih.gov/nuccore/, KY858944.
